# Physicians' Ability to Predict Hospital Length of Stay for Patients Admitted to the Hospital from the Emergency Department

**DOI:** 10.1155/2012/824674

**Published:** 2012-01-26

**Authors:** Gregory Mak, William D. Grant, James C. McKenzie, John B. McCabe

**Affiliations:** ^1^Department of Emergency Medicine, State University of New York Upstate Medical University, Syracuse, NY 13210, USA; ^2^Jefferson Medical College, Thomas Jefferson University, Philadelphia, PA 19107, USA

## Abstract

Accurate predictions of patient length of stay (LOS) in the hospital can effectively manage hospital resources and increase efficiency of patient care. A study was done to assess emergency medicine physicians' ability of predicting the LOS of patients who enter the hospital through the ER. Results indicate that EM physicians are relatively accurate with their pediatric patients than any other age groups. In addition, as actual hospital LOS increases, the prediction accuracy decreases. Possible reasons may be due increasing medical complications associated with increasing age and this may lead to overall longer stays. Other variables such as the admitted service of the patient are not statistically significant in predicting LOS in this study. Future studies should be done in order to determine other variables that may affect LOS predictions.

## 1. Introduction

The ability to reasonably predict the length of stay (LOS) for patients admitted from the emergency department is important in determining and managing healthcare resources. To optimize and effectively address patient care, consistent discrepancies between predicted and actual LOS may strain resources and cause consequences that can burden both the patient and the hospital. Admittedly, determining with high accuracy is difficult as estimates are compounded by multiple variables such as patient care, insurance, and morbidity, all of which can influence LOS [1]. Previous efforts have been made to quantify predicted LOS and compare it with the actual patient LOS in specific types of cases such as surgery and trauma. These cases, however, tend to have proven and well-studied protocols and neural networks that dictate patient flow during admittance while also predicting LOS [2]. Psychiatry also has extensive literature documenting LOS prediction. Data from the psychiatric literature have shown that certain variables available during patient admittance are able to be assessed in predicting patient LOS [3].

Despite the breadth of research done with LOS and specific fields such as surgery and psychiatry, very little information exists regarding LOS prediction through emergency department admittance. This study attempts to address this gap in knowledge by assessing the accuracy of emergency medicine physicians in their prediction of the LOS of patients being admitted to the hospital. As many patients continue to be admitted through the hospital via the emergency department and those patients still account for a significant portion of hospitalizations, it is important that emergency medicine physicians provide an accurate estimate [4]. Accurate LOS predictions aid in directing patient flow by indicating inefficiencies in the hospital systems and assessing the need for improvement [5]. An accurate LOS estimate may also affect the care and emotional well-being of both the patient and his caretakers.

## 2. Materials and Methods

Physicians in the emergency department of a level 1 trauma university hospital (annual volume 55,000) were asked to identify patients who were scheduled for hospital admission. The emergency department has separate adult and pediatric services. Patient demographic information was recorded along with date of emergency department admission, patient age, presenting complaint, and physician diagnosis. The admitted service was also recorded. The patient's current attending emergency department physician was asked to predict the patient's length of stay in the hospital based upon the physician's personal experience. Patients in the study were then followed up to determine the actual LOS in the hospital per hospital records. The patient diagnosis in the hospital along with additional diagnosis that was given was also recorded. All collected data information was then recorded on Microsoft Excel secure spreadsheets and analyzed using IBM-SBSS version 18 software. This study was approved by SUNY Upstate's Institutional Review Board.

## 3. Results and Discussion

A total of 431 of 492 (87.6%) of the patients had sufficient data to analyze length of stay predictions. The overall average predicted length of stay is 3.86 days (SD ± 2.77 days), while the average actual LOS is 5.62 days (SD ± 7.71 days). Overall the difference between the two LOS means is statistically significant with a mean difference of −1.76 days (SD ± 7.55 days, 95% CI −2.47 to −1.04) *t* = 4.82, *P* < 0.00 (Table 1).

There is no statistically significant difference of predicted and actual LOS between the admitted services (Table 2). A univariate analysis of the data by admitted services and age group also showed no statistically significant difference amongst the groups.

Bland-Altman analysis was undertaken to assess the size of the difference between predicted and actual length of stay. Bland-Altman plots the average of two measurements against their difference. Values which lie between ±1.96 SD of the mean are considered to be equivalent [6, 7]. For all patients the mean difference in prediction was (−0.05 SD 2.7, 95% CI −0.33 to 0.22). The prediction limits for all patients were upper limit (UL) = 5.31 and lower limit (LL) = −5.42. For patients less than 18 years the mean difference was 0.44, (SD 1.67, 95% CI −0.11 to 0.95, UL = 3.71, LL = −2.86), for patients between 18 years and 65 years, the mean difference was −0.21 (SD 2.96, 95% CI −0.59 to 0.17  UL = 5.61, LL = −6.02), and for those over age 65 the mean difference was −1.06 (SD 3.89, 95% CI −1.82 to −0.30, UL = 6.57, LL = −8.70).

Table 3 shows the diagnoses made of the 10% of the group with the shortest length of stays in the hospital and those of the 10% with the longest length of stays. As evidenced by the diagnosis type, the shortest length of stays has a majority of uncomplicated or less severe cases. The longest length of stays, however, reveals a variety of complex medical conditions such as septicemia and disordered urea cycle metabolism.

## 4. Conclusion

 An analysis of emergency medicine physician's accuracy in predicting the length of stay of patients admitted to the hospital from the emergency department revealed significant statistical differences overall. Despite this difference, the accuracy of the physician's prediction of all cases sampled is fairly high, with their prediction differing generally by less than two days (1.76 days). Further analysis of the data revealed that age group is the most predominant LOS accuracy predictor of emergency medicine physicians. The range of the prediction boundaries for pediatric patients 6.56 (UL = 3.71, 95% CI 2.78 to 4.62 and LL = −2.86, 95% CI −3.78 to −1.93) was the smallest of the group comparisons where the range for those between 18 and 65 was 11.63 (UL = 5.60, 95% CI 4.95 to 6.25 and LL = −6.02, 95% CI −6.67 to −5.37) and for those aged 65 and older (UL = 6.57, 95% CI 5.27 to 7.87 and LL = −8.70, 95% CI −10.0 to −7.40). In this study, the physicians were able to more accurately predict the LOS of pediatrics patients compared to the other two age groups of adults and the elderly. As actual length of stay increases, the difference between prediction and actual also increases seen in the Bland-Altman plots. This is likely due to the increased medical complexity of some patients making even inpatient prediction of length of stay problematic. Physicians' ability to predict pediatric LOS is closer to actuality likely because of the relative lack of pediatric patients with complex medical issues. These patients are likely to have focused medical issues.

**Figure 1:**
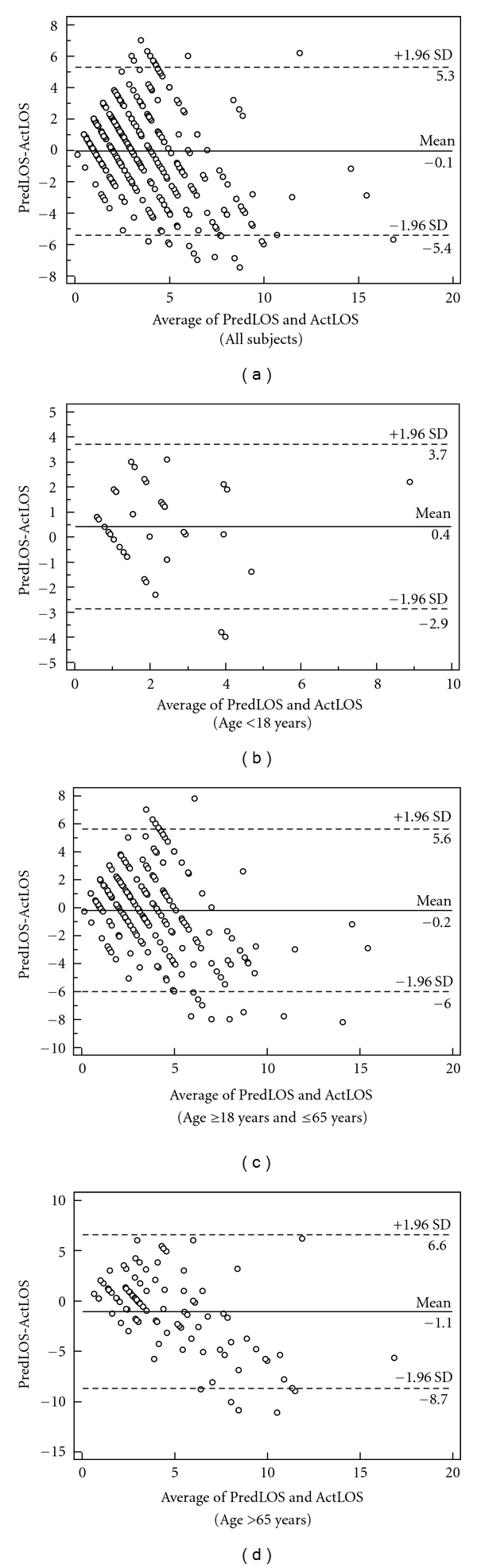
Bland-Altman analysis of predicted and actual LOS differences.

As can be seen in the total sample plot (Figure 1(a)), when actual LOS is about 5 days or less, the physicians' predictions are fairly close to actual LOS. As actual length of stay increases, implying patients with more complex medical issues, the physicians' ability to predict becomes less certain. Predictions for pediatric patients are generally very good. Predictions for adult and elderly patients become less accurate as the actual length of stay increases.

 When the difference between actual and predicted LOS was divided into categories in terms of admitting services of the patients, there were no statistically significant differences between the four major groups of neurology, psychiatry, surgery/trauma, and medicine. However, descriptive statistics of the data suggests that surgery/trauma has the lowest average difference out of all the categories. As mentioned earlier, this may be due to the extensive evaluations that surgery cases go through in terms of predicting LOS amongst surgeons. This information and experience may also be available or known for emergency medicine physicians and can therefore be a reason for the smaller difference in the predicted and actual LOS. Psychiatry, on the other hand, had the highest difference between the actual and predicted LOS. A reason for this may be that psychiatric patients admitted to the hospital may have unpredictable or behavioral manifestations that can influence the length of stay in the hospital that emergency medicine physicians may be unaware of or had not taken notice [8]. Further studies evaluating and assessing predicted LOS in patients regarding admitted services in hospitals should be done to accurately portray the importance of the type of admitted services in affecting LOS predictions.

Major limitations of this study included a lack of information on the severity of cases. The data collected represented a general view of all patients who come into the emergency room. However, aside from the admitted service to the hospital, there is little information on the patient's condition and its severity. These factors may affect LOS prediction and can also introduce bias in the physician's judgment and evaluation.

 While it is improbable for physicians to accurately predict the length of stay of patients in hospitals, providing an accurate estimate allows for better management of resources and hospital beds. This study was intended to assess the accuracy of LOS predictions of emergency medicine physicians since a large number of patients admitted to hospitals enter via the emergency department. Although the overall accuracy is fairly high, physicians are more capable of accurately predicting pediatric LOS cases compared to adult or the elderly. In addition, although not statistically significant, there is an indication that physicians are more accurate in predicting LOS in patients that require surgery or neurology than psychiatry or medicine. This may indicate the need for better assessment and evaluation for the complicated natures of psychiatric patients and patients that require medical interventions.

## Figures and Tables

**Table 1:** Patient data by the separate age groups with the mean, SD, and 95% CI.

	*N*	Mean difference (actual-predicted LOS in days)*	SD (days)	95% CI
Pediatrics (<18)	47	1.70	±1.76	1.18–2.22
Adult (18–65)	262	3.62	±5.22	2.98–4.25
Elderly (>65)	122	5.35	±9.74	3.61–7.10
Total	431	3.90	±6.69	3.27–4.53

*F = 5.77, *P* < 0.003.

**Table 2:** Predicted and actual LOS within admitting services.

	*N*	Mean difference (actual-predicted LOS in days)*	SD (days)	95% CI
Neurology	41	3.64	±4.16	2.33–4.96
Psychiatry	32	4.42	±6.32	2.14–6.70
Surgery/trauma	81	3.31	±3.96	2.44–4.19
Medicine	275	3.93	±7.38	3.05–4.81
Total	429	3.82	±6.51	3.21–4.44

*F = 0.29, *P* < 0.832.

**Table 3:** Top 10% shortest and longest length of stay diagnosis.

Top 10% shortest LOS diagnosis	Count	Top 10% longest LOS diagnosis	Count
Abnormal involuntary movement	1	Bipolar disorder	1
Anal fissure	1	Cellulitis (foot)	1
Anomalies (spleen)	1	Cerebral artery infarction	1
Cellulitis (buttocks)	1	Chest pain	1
Cellulitis (face)	1	Congestive heart failure	1
Cerebral artery infarction	1	Convulsions	1
Chest pain	4	Chronic obstructive asthma	1
Chronic pain	1	Disordered urea cycle metabolism	1
Chest wall contusion	1	Diabetes type 2 w/complications	3
Coronary artery disease	1	Encephalitis	1
Diabetes type 1 w/complications	1	Fall	3
Eye swelling/mass	1	Fistula (persistent post-op)	1
Failure to thrive	1	HIV	1
Fall	3	Idiopathic neuropathy	1
Fetal jaundice	1	Intracerebral hemorrhage	1
Fever	2	Motor vehicle collision	1
Gall bladder calculi	1	Neurohypophysis	1
Intracerebral hemorrhage	1	Poison (antihypertensive agent)	1
Laryngeal spasm	1	Psychosis	1
Lumbosacral neuritis	1	Psychosis (severe recurrent)	1
Ovarian cyst	1	Rheumatic heart failure	1
Poison (exhaust gas, anesthesia)	2	Septicemia	2
Riding animal accident	1	Subarachnoid hemorrhage	1
Skin sensation disturbances	1	Syncope + collapse	2
Syncope and collapse	1	Trauma (falling object)	1
Tietze's disease	1		
Transcerebral ischemia	1		
Urinary tract infection	1		
Vertigo	1		
